# Bisphosphonates as Potential Inhibitors of Calcification in Bioprosthetic Heart Valves (Review)

**DOI:** 10.17691/stm2022.14.2.07

**Published:** 2022-03-28

**Authors:** T.P. Timchenko

**Affiliations:** Junior Researcher, Laboratory of Bioprosthetics; Meshalkin National Medical Research Center of the Ministry of Health of the Russian Federation, 15 Rechkunovskaya St., Novosibirsk, 630055, Russia

**Keywords:** bisphosphonates, aminobisphosphonates, calcification, anticalcium agents, bioprosthetic heart valves

## Abstract

As early as 50 years ago, bisphosphonates turned from a water treatment agent into one of the most widely used groups of drugs for the treatment of various diseases of calcium metabolism (bone tissue resorption, oncological complications of neurodegenerative diseases and others). Years of research on bisphosphonates have contributed to the understanding of their molecular and cellular pathways of their action. All bisphosphonates have a similar structure and common properties, however, there are obvious chemical, biochemical, and pharmacological differences between them. Each bisphosphonate has its own unique profile. This review summarizes data on the mechanisms of action of bisphosphonates, demonstrates the experience and prospects for their use for the modification of cardiovascular bioprostheses, since the issue of preventing bisphosphonate calcification has not been settled yet.

## Introduction

Bisphosphonates (BP), or diphosphonates, as called before, have been known for a long time. They were first synthesized by German chemists as early as 1865 [1, 2], but they have been used for the treatment of calcium metabolism disorders only in the last 50 years. Currently, BPs are one of the most widely used groups of drugs for the treatment of Paget disease, osteoporosis, breast cancer and neoplastic bone metastases, multiple myeloma, some other rare bone diseases, neurodegenerative diseases, and also in dentistry [3-11]. In veterinary medicine, these drugs are used to solve the same problems in the treatment of different animal species [12]. In addition, bisphosphonates are used for targeted delivery of drugs to the bone: antibiotics, hormones, and anticancer drugs [13]. Since 1970, BPs have been used as radioactively labeled drugs in the diagnosis of skeletal diseases [14]. The possibility of using Zoledronate BP as an immunomodulator in the complex treatment of pneumonia caused by SARS-CoV-2 is being considered [15].

The discovery of the BPs’ biological effects goes back to the study of the mechanisms of calcification and the role of pyrophosphate in it. As early as the 1930s, polyphosphates were found to act as natural physiological regulators of the calcification process due to their ability to inhibit the deposition of calcium salts. In the 1960s, it was found that biological fluids (urine and blood plasma) contain a substance that inhibits the precipitation of calcium phosphate, namely pyrophosphate [16, 17]. It has a high affinity for calcium crystals, slows down their formation and dissolution *in vitro*, and inhibits calcification *in vivo*, but when taken orally, it is rapidly metabolized in the body due to hydrolysis in the gastrointestinal tract [18, 19]. The search for compounds resistant to enzymatic hydrolysis and similar to pyrophosphate in terms of physical and chemical activity led researchers to BPs.

## Molecular structure and pharmacological efficacy of bisphosphonates

**Figure 1.**
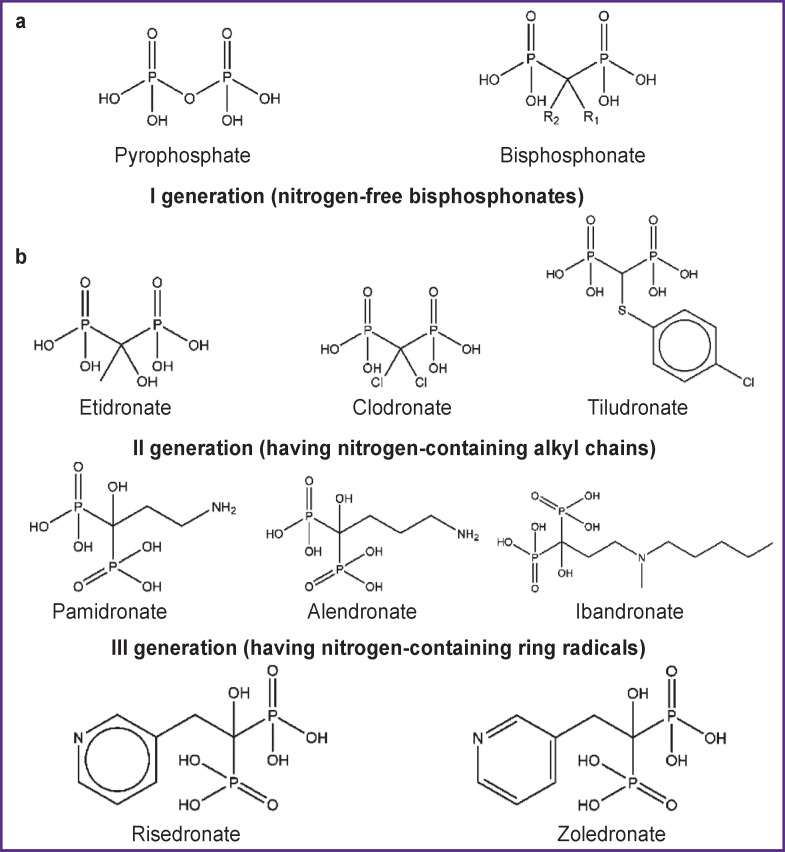
Molecular structure of pyrophosphate and bisphosphonate (a) and structural formulas of some bisphosphonates (b)

Bisphosphonates are synthetic analogues of pyrophosphate with two phosphonate groups bound to a central carbon atom. The P–C–P group in the BP structure makes them resistant to enzymatic hydrolysis, in contrast to the hydrolytically unstable P–O–P bond in the pyrophosphate structure. Besides, BPs have two additional side chains in a molecule, which are absent in pyrophosphate. They are called R1 and R2, respectively, and are also bound to the central carbon atom (Figure 1) [20-24].

Bisphosphonates bind with hydroxyapatite due to chelation of calcium ions on the surface of apatite crystals by two phosphonate groups located in close proximity, which leads to the formation of a bidentate bond [25-28].

The type of side chains in BPs is an important factor determining their properties. Hydroxyl substitution in R1 was found to increase the BPs’ affinity for calcium crystals due to the formation of a tridentate bond [29]. It is the hydroxyl group that the majority of clinically used BPs contain in the R1 position. BPs with R1 substituted for Cl^–^ or H^+^ ions (Clodronate and Tiludronate) provide bidentate binding to calcium crystals and have a significantly lower binding affinity [30, 31]. The configuration of the R2 side chain determines the antiresorptive activity of BPs with respect to bone tissue [32, 33]. On the whole, the presence of R1 and R2 side chains makes it possible to introduce numerous substitutions and synthesize a large number of substances with different properties.

According to the chemical structure of R2, BPs are subdivided into nitrogen-free and nitrogen-containing compounds (see Figure 1). The nitrogen atom in the structure has an impact on the antiresorptive efficacy of nitrogen-containing BPs, increasing it by 10–10,000 times relative to nitrogen-free ones (see the Table).

**Table:** Bisphosphonates used in clinical practice and their relative antiresorptive activity

Group	International non-proprietary name	Trade name of the drug	Antiresorptive potential*	Administration mode	Mechanism of action
I generation (nitrogen-free)	Etidronic acid	Xidiphone	1	Orally	Metabolized intracellularly (in osteoclasts) to cytotoxic adenosine triphosphate analogues
		Pleostat		Intravenously	
		Didronel			
		Didrocal			
		Etidrocal			
		Etiron			
	Clodronic acid	Benefos	1–10	Perorally	
		Sidronate		Intravenously	
		Klobir			
		Clasteon			
	Tiludronic acid	Skelide	10	Perorally	
II generation (with nitrogen-containing alkyl chains)	Pamidronic acid	Aredia	100	Intravenously	They inhibit the enzyme farnesyl diphosphate synthase, resulting in a decrease in the formation of mevalonate, a substance necessary to maintain osteoclastic vital activity
	Alendronic acid	Tevanat	100–1000	Perorally	
		Fosamax			
		Fosavance			
		Adrovance			
		Act Alendronate			
		Bimisto			
	Ibandronic acid	Bandronat	1000–10,000	Perorally		
		Boniva		Intravenously		
		Bonviva				
		Iasibon				
III generation (with nitrogen-containing ring radicals)	Risedronic acid	Risendros	1000–10,000	Perorally	They inhibit the enzyme farnesyl diphosphate synthase, resulting in a decrease in the formation of mevalonate, a substance necessary to maintain osteoclastic vital activity
		Actonel			
	Zoledronic acid	Zoledronate	>10,000	Intravenously	
		Aclasta			
		Zometa			
		Reclast			

* the antiresorptive efficacy of nitrogen-containing bisphosphonates relative to nitrogen-free ones is indicated.

The presence of a positively charged R2 group enables BPs to bind to the mineral surface of the bone, which subsequently increases the affinity of hydroxyapatite for negatively charged phosphonate groups as a result of electrostatic interactions [34]. Another important factor in the higher activity of nitrogen-containing BPs compared to nitrogen-free ones is the formation of hydrogen bond between the BP amino group and the hydroxyapatite surface. Alendronate with a free amino group is an example of this bond [35]. This explains the strong affinity of amine-containing BPs for bone tissue and their use in the treatment of bone diseases [36-39]. Moreover, BP binding with carbonate apatite has been reported [36-39]. This proves the influence of the R2’s structure on the absorption, distribution, and long-term deposition of BPs in bone tissue.

The BPs of the first generation differ from other groups by the absence of nitrogen in their composition (nitrogen-free bisphosphonates). The scope of effects of these substances is narrower than that of aminobisphosphonates. Nevertheless, the treatment and prevention of various diseases associated with bone resorption with these drugs has proven to be highly effective. BPs of the first generation succeed in the correction of hypercalcemia, prevention efforts — to prevent the development of bone metastasis in certain cancers, Paget disease, and in the treatment of osteoporosis (but it is contraindicated in juvenile osteoporosis) [7, 40-43].

Amino-containing BPs of the second generation are characterized by a wider scope of actions and higher efficiency. Thus, for example, Pamidronate has proven to be effective in the treatment of patients with multiple myeloma and breast cancer with bone metastases, i.e. tumors characterized primarily by the development of osteolytic metastases [16, 44].

Despite the proven dose-dependent effect of Pamidronate, its high doses are practically not used due to adverse effects on the gastrointestinal tract [45, 46]. In patients with tumor-induced hypercalcemia, Pamidronate has exhibited an advantage over Clodronate, primarily, in the duration of normocalcemia, since the average duration of the effect of Clodronate is 14 days compared to 28 days for Pamidronate [47]. Aminobisphosphonates can also be used to prevent complications of bone metastasis. The results of studies with Clodronate and Pamidronate revealed a significant reduction in the incidence of complications with prolonged use of Pamidronate [38].

The development of new third-generation BPs with a reduced frequency of administration (once per week or once per month) contributed to a significant increase in adherence to treatment, optimization of therapy outcomes, and a reduction in adverse events. Thus, zoledronic acid, which has the highest affinity for bone tissue hydroxyapatite in comparison with other aminobisphosphonates, such as Alendronate, Ibandronate, Risedronate, provides a greater therapeutic effect and less side effects [48-53].

Nancollas et al. [54] determined the kinetic ability of BPs’ binding in their studies. They found that Clodronate was the weakest inhibitor of the growth rate of hydroxyapatite and had the lowest kinetic affinity constant. Other authors [7, 22] found differences between hydroxyl-substituted bisphosphonates and ranked them according to hydroxyapatite binding affinity as follows: Zoledronate > Alendronate > Ibandronate > Risedronate > Etidronate > Clodronate.

## Cellular and molecular mechanisms of bisphosphonate action

Together with synthesizing more powerful BPs, it has become obvious that their biological activity is not explained by physical and chemical properties alone. This stimulated studies of the mechanisms of BPs’ action at the cellular level [3, 55-59].

The cellular mechanisms of action of BPs are based on the inhibition of bone tissue resorption due to their selective binding and adsorption on the bone mineral surface. Having a high affinity for calcium ions, they perfectly penetrate the bone tissue. There, BP molecules are concentrated around osteoclasts, creating a high concentration in resorption lacunae. *In vitro* studies have shown that BPs influence the depth of resorption lacunae, reducing it. Within osteoclasts, they initiate a number of changes that reduce the ability of bone tissue to resorb (loss of brush border, cytoskeleton destruction, inability of osteoclasts to move or bind to bone tissue). After BPs bind to osteoclasts, they impair their biochemical processes, causing apoptosis [34, 60].

At the molecular level, the biochemical mechanisms of BP action also differ and depend on their structure. There are two major mechanisms of their action.

Nitrogen-free BPs of the first generation behave like pyrophosphate analogs: they are involved in the metabolism of stable ATP analogs (to adenosine-5’-(β,γ-dichloromethylene)-triphosphate) due to the action of aminoacyl-tRNA synthase [42]. Intracellular accumulation of these non-hydrolysable metabolites in osteoclasts causes a deficiency of functional ATP and also inhibits the mitochondrial ADP/ATP translocase, which, in turn, leads to osteoclast apoptosis [61-63].

Highly active nitrogen-containing II generation N-BPs are not metabolized, but directly induce osteoclast apoptosis by inhibiting the biosynthesis of mevalonate, which is involved in the formation of cholesterol and isoprenoid lipids, including isopentenyl pyrophosphate (IPP), farnesyl pyrophosphate (FPP), and geranylgeranyl pyrophosphate (GGPP). However, the main target of this group of BPs is farnesyl pyrophosphate synthase (FPPS), one of the enzymes involved in the metabolism of pyrophosphate-containing isoprenoid lipids [23, 39, 64]. FPP and GGPP are required for post-translational prenylation of small G proteins such as Rab, Rac, Ras, and Rho. These key G proteins, prenylated at a cysteine residue, regulate various cellular processes of osteoclast function, those of maturation and survival. Therefore, inhibition of FPPS leads to loss of resorption capacity of osteoclasts or inhibits osteoclastogenesis [36, 65, 66]. The ability to inhibit the process of protein modification in osteoclasts leads to apoptosis of mature cells, which is proved by the appearance of specific changes in the cell and structure of the nucleus [67]. At the same time, osteoclast precursor cells lose their ability to differentiate and mature, which naturally leads to a decrease in the number of osteoclasts [28]. Moreover, *in vitro* data indicate that, under the influence of BP, osteoblasts reduce the secretion of the osteoclast-stimulating factor [68]. BPs are capable of recycling, i.e. return to the systemic circulation from the bone surface resorbed by osteoclasts. BP molecules released from the bone tissue can attach to another part of the bone. Continuous BP administration increases the “bisphosphonate load” on the bone, which determines the unique feature of this class of drugs — the preservation of the clinical effect for a long time after discontinuation of therapy [29, 69-72].

The mechanism of BP action is partially similar to the mechanism of action of statins, since they also inhibit the enzymes involved in the of mevalonate metabolism, though, statins participate only in one of the first stages, inhibiting HMG-CoA reductase [67].

**Figure 2.**
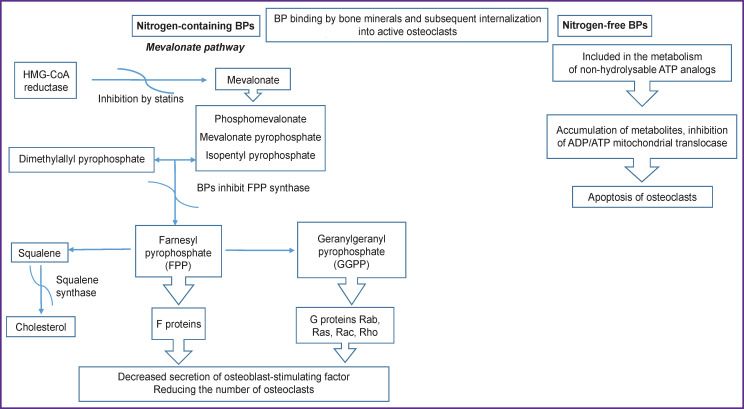
Cellular and biochemical mechanism of action of bisphosphonates

Thus, the mechanism of BP action is based on a triple effect on the key processes of bone remodeling: physical and chemical binding to hydroxyapatite, a direct effect on the resorption activity of osteoclasts, and stimulation of new bone formation (Figure 2).

## The use of bisphosphonates for the modification of cardiovascular bioprostheses

The biological prostheses for the correction of cardiovascular diseases have been used for more than 60 years [73-75]. Various xenogenic materials are used for the production of valve and vascular prostheses: the porcine aorta, aortic valve, and pericardium, as well as the bovine pericardium, jugular vein, and internal thoracic artery. These materials differ in microstructure, ratio of fibrillar proteins and amino acids. Since 1967, glutaraldehyde (GA) has been used in the production of biological prostheses for tissue preservation [76-79]. GA provides a high density of collagen cross-linking and significantly increases its resistance to the action of proteolytic enzymes. At the same time, bioprosthetic materials treated with GA acquire a marked tendency to pathological calcification [80-83].

According to modern concepts, the calcification of bioprosthetic tissue is based on the structural features of the chemical bonds between collagen and GA. The formation of cross-links occurs mainly due to the reaction of ε-amino groups of lysine and hydroxylysine with polymeric GA. These bonds contain several active oxygen atoms capable of forming strong complexes with calcium cations. Calcification can be provoked by the bond of polymerized GA molecules, which is similar to pyridine bases found in bone tissue collagen [84-86], a degree of mineralization being directly dependent on the density of cross-links in the collagen matrix. In addition, the level of glycosaminoglycans and proteoglycans cross-linked with collagen and preventing spontaneous precipitation of calcium salts in soft tissues decreases in the biomaterial during conservation [87-89].

For many years, researchers have been studying the mechanisms of calcification and searching for new methods for the conservation of biological tissue [90-96]. One of the avenues of investigation is related to drugs of the BP group. Thus, systemic parenteral administration of etidronic and pamidronic acids during subcutaneous implantation of the biomaterial in rats provided 97% inhibition of calcification. But the doses of the administered drug in these experiments significantly exceeded the therapeutic ones, which caused complications, such as osteomalacia and calcium imbalance. Long-term use of these drugs in experimental animals impaired general somatic growth, and short-term therapy was ineffective [97].

To avoid complications associated with the systemic use of BPs, the study of methods of local therapy began. The first such experience was gained by using polymer matrices that provide controlled release of the drug. The biomaterial and the polymer matrix were implanted in immediate proximity (thus, systemic adverse effects were avoided), but the matrix was depleted rather quickly, which made it impossible to create long term therapeutic BP concentration [98-101]. Recently, a method of local application (transcatheter delivery) of Zoledronate has been proposed to prevent calcification of the aortic valve with the development of aortic stenosis in experimental animals [102]. The study was conducted on a small group of New Zealand rabbits with highly pronounced aortic stenosis. A medicinal composition with 500 μg/L Zoledronate was used as an anticalcium therapy. It was applied directly to the valve leaflets. The experiment was completed after 28 days. Histological examination of the leaflets demonstrated a significant reduction in the area of calcium lesions by almost 40% in the group treated with Zoledronate, compared with the control group. Despite good results, this technique is rather complicated, and most authors still recommend the systemic use of BPs in the clinical treatment of aortic stenosis and aortic valve calcification [103-106].

A step forward was using the method of immobilization of BP molecules on biological tissues [107]. It was first described by Fleisch et al. as early as 1968 [108]. At the end of the last century, numerous papers were published confirming the anticalcium effect of BPs immobilized on collagen biomaterials crosslinked with GA [109-116].

The nitrogen atom in R2 of the amine-containing BP molecule can covalently bind with the free groups of the bifunctional preservative which remain after the completion of the cross-linking process (masking group) [114]. However, the primary, secondary, and tertiary nitrogen atoms in the amino group have different binding reactivity with aldehyde groups. Historically, the first and most well studied for the purpose of anticalcium modification is Pamidronate [111]. In addition, other compounds of this group were also studied [38, 112-119]. It has been established that not all BPs have the same anticalcifying effect. The structure of BPs and the presence of free phosphonic groups after immobilization on the biomaterial determine their biological and calcium-inhibiting activity. At the same time, there is no correlation between the calcium-inhibiting activity and the amount of the drug fixed on the biomaterial [120]. Pamidronate demonstrated the highest calcium-inhibiting activity on immobilization on the GA-treated biomaterial [120].

It should be noted that different BPs at the same concentration of working solutions are immobilized on GA-treated biological tissues in different amounts. The amount of immobilized BP depends on its structure as well as on the species and tissue affiliation of the material [121-123]. No relationship was found between the amount and effectiveness of BP associated with the preserved material. It is interesting to note that Zoledronate, which has the highest systemic efficacy among all known BPs, had the least anticalcium effect in the immobilized state [120]. Russell et al. [34] suggest that some BP molecules bind to residual aldehyde groups, while others bind directly to proteins, forming hydrogen bonds (similar to the interaction of BPs with Thr or Lys FPPS [36]) with amino acids that can potentiate calcification. In both cases, the phosphonic groups remain free and can affect mineralization due to the direct physical and chemical binding of hydroxyapatite. The study established [120] that, when developing a strategy for modifying biomaterials with immobilized BPs, it is necessary to take into account the whole set of factors, the main of which are the molecular structures of BP itself, the preservative, and the predominant protein of the connective tissue matrix. Their main components are collagen and elastin, consisting of soluble tropoelastin molecules, bound by desmosine and isodesmosine, which form insoluble elastin [120]. GA pericardial cross-linking has been shown previously [122, 123] to stabilize collagen but not elastin, which can cause elastin degradation and, hence, a decrease in the elastic properties of the tissue. This occurs mainly due to very few free amino groups in elastin that are needed for crosslinking. All the GA-treated biomaterials have a high calcium-binding capacity (>100 μg/mg dry tissue). Preservation with diglycidyl ether of ethylene glycol (DEE) reduces the calcium level in the wall of the vein and pericardium by 4 to 40 times, respectively, but does not affect the wall of the aorta. Mineralization in the walls of the aorta and vein treated with GA and DEE is predominantly associated with elastin. Thus, it can be hypothesized that improved elastin stabilization would reduce calcification and increase tissue durability. BP modification reduces elastin calcification, but does not completely block it. The search for an “ideal” cross-linking agent for a biomaterial goes on. Each xenogenic material requires an individual protection strategy [124-126].

## Conclusion

Over the half-century history of the medical use of bisphosphonates, plenty of new compounds with various groups in the R1 and R2 positions and, accordingly, having different anticalcium activity have been synthesized. Researchers have a choice among existing drugs and ample opportunities for the synthesis of new ones. It is necessary to expand the indications for the use of bisphosphonates, especially for immobilization on xenogenic bioprosthetic materials in order to prevent their calcification in the recipient’s body. This area has not yet been sufficiently studied since the causes for the tissue specificity of various bisphosphonates, the peculiarities of their binding, and effectiveness depending on the crosslinking agent used for preservation, as well as the mechanisms of the anticalcifying action of immobilized bisphosphonates, are unknown.

There is still no unified modification method for different bisphosphonates and various tissues. However, the accumulated experience indicates that the prospects for using bisphosphonates as an anticalcium agent for the creation of cardiovascular bioprostheses are quite real, but this problem needs further investigation.
